# Closing the Gap: Ensuring Minimum Required Physical Health Equipment Is Available on Inpatient Wards: An Audit

**DOI:** 10.1192/bjo.2026.11805

**Published:** 2026-06-30

**Authors:** Hasan Obaid, Melissa Capraro, Huw Evans, Gurbinder Singh Maumi

**Affiliations:** Midlands Partnership NHS Foundation Trust, Shrewsbury, United Kingdom

## Abstract

**Aims:**

To identify mental health inpatient wards that may not have the full range of physical health monitoring equipment in line with the **Royal College of Psychiatrists (RCPsych) 2009 standards**, and to highlight any gaps or deficiencies so they can be addressed appropriately as physical examination and physical examination monitoring are essential to all patients in psychiatry.

**Methods:**

The audit was conducted at The Redwoods Centre, including the acute adults, older adults and the forensic wards. Community mental health services in Shropshire were excluded. The sample comprised seven inpatient wards, each containing a designated clinic room. The data was collected in August 2025. The audit tool consisted of a checklist based on *Physical Health in Mental Health: Final Report of a Scoping Group (Royal College of Psychiatrists).* The compliance standard was set to 100%. The data collection for the first audit cycle was completed within 1 a day. A re-audit to close the loop was planned for 6 months after the first cycle. The audit process and results were monitored through the South Staffordshire & Worcestershire (ST&W) Quality & Safety Sub-Committee. Results will be shared with South Staffordshire and Forensic Quality/Audit *Committees* colleagues for information.

**Results:**

All wards demonstrated greater than 80% compliance with required physical health monitoring equipment. Overall compliance across wards was 87%. Equipment such as alcometers, Snellen charts, ophthalmoscopes, and otoscopes were not permanently available on all wards but were borrowed from other wards as required. Neurological testing pins were absent on most wards, with the exception of one of the wards. Existing arrangements for sharing equipment between wards were reviewed and formally confirmed. Plans are in place to procure missing equipment where necessary to improve compliance and reduce reliance on borrowing.

**Conclusion:**

All inpatient wards at The Redwoods Centre met a **compliance threshold of 80%**, with an overall compliance rate of **87%** against RCPsych physical health monitoring equipment standards. While some essential items were not consistently available on all wards, interim measures such as equipment sharing were in place. The absence of neurological testing pins on most wards was identified as a key area for improvement. Planned procurement and formalised sharing agreements aim to address these gaps ahead ofthe re-audit cycle.

